# Stabilization of Silver Nanoparticles on Polyester Fabric Using Organo-Matrices for Controlled Antimicrobial Performance

**DOI:** 10.3390/polym14061138

**Published:** 2022-03-12

**Authors:** Ana Isabel Ribeiro, Vasyl Shvalya, Uroš Cvelbar, Renata Silva, Rita Marques-Oliveira, Fernando Remião, Helena P. Felgueiras, Jorge Padrão, Andrea Zille

**Affiliations:** 1 Centre for Textile Science and Technology (2C2T), Department of Textile Engineering, University of Minho, Campus of Azurém, 4800-058 Guimaraes, Portugal; afr@2c2t.uminho.pt (A.I.R.); helena.felgueiras@2c2t.uminho.pt (H.P.F.); padraoj@2c2t.uminho.pt (J.P.); 2 Department of Gaseous Electronics (F6), Jožef Stefan Institute, SI-1000 Ljubljana, Slovenia; vasyl.shvalya@ijs.si (V.S.); uros.cvelbar@ijs.si (U.C.); 3 Faculty of Mathematics and Physics, University of Ljubljana, SI-1000 Ljubljana, Slovenia; 4 Associate Laboratory i4HB—Institute for Health and Bioeconomy, Faculty of Pharmacy, University of Porto, 4051-401 Porto, Portugal; rsilva@ff.up.pt (R.S.); ritoliveira.m@gmail.com (R.M.-O.); remiao@ff.up.pt (F.R.); 5 UCIBIO—Applied Molecular Biosciences Unit, REQUIMTE, Laboratory of Toxicology, Department of Biological Sciences, Faculty of Pharmacy, University of Porto, 4051-401 Porto, Portugal

**Keywords:** silver nanoparticles, chitosan, hexamethyldisiloxane, antimicrobial textiles, spray deposition

## Abstract

Antimicrobial textiles are helpful tools to fight against multidrug-resistant pathogens and nosocomial infections. The deposition of silver nanoparticles (AgNPs) onto textiles has been studied to achieve antimicrobial properties. Yet, due to health and environmental safety concerns associated with such formulations, processing optimizations have been introduced: biocompatible materials, environmentally friendly agents, and delivery platforms that ensure a controlled release. In particular, the functionalization of polyester (PES) fabric with antimicrobial agents is a formulation in high demand in medical textiles. However, the lack of functional groups on PES fabric hinders the development of cost-effective, durable systems that allow a controlled release of antimicrobial agents. In this work, PES fabric was functionalized with AgNPs using one or two biocompatible layers of chitosan or hexamethyldisiloxane (HMDSO). The addition of organo-matrices stabilized the AgNPs onto the fabrics, protected AgNPs from further oxidation, and controlled their release. In addition, the layered samples were efficient against *Staphylococcus aureus* and *Escherichia coli*. The sample with two layers of chitosan showed the highest efficacy against S. aureus (log reduction of 2.15 ± 1.08 after 3 h of contact). Against *E. coli*, the sample with two layers of chitosan showed the best properties. Chitosan allowed to control the antimicrobial activity of AgNPs, avoid the complete loss of AgNPs after washings and act in synergy with AgNPs. After 3 h of incubation, this sample presented a log reduction of 4.81, and 7.27 of log reduction after 5 h of incubation. The antimicrobial results after washing showed a log reduction of 3.47 and 4.88 after 3 h and 5 h of contact, respectively. Furthermore, the sample with a final layer of HMDSO also presented a controlled antimicrobial effect. The antimicrobial effect was slower than the sample with just an initial layer of HMDSO, with a log reduction of 4.40 after 3 h of incubation (instead of 7.22) and 7.27 after 5 h. The biocompatibility of the composites was confirmed through the evaluation of their cytotoxicity towards HaCaT cells (cells viability > 96% in all samples). Therefore, the produced nanocomposites could have interesting applications in medical textiles once they present controlled antimicrobial properties, high biocompatibility and avoid the complete release of AgNPs to the environment.

## 1. Introduction

Textiles may provide an excellent environment for microorganisms to thrive, presenting a suitable availability of nutrients, moisture, oxygen, and favorable temperature ranges. The functionalization of textiles with antimicrobial finishing agents has been widely applied to achieve technical materials and avoid the deterioration caused by microorganisms [1]. Increased healthcare awareness about hygiene and health issues has extended the global market to the antimicrobial textiles field. These textiles may be employed in several applications (e.g., wounds, sutures, and tissue engineering products) to prevent microbial proliferation and, hence, bad odors, stains, infections, a reduction in the textile’s mechanical properties, and cross-contamination [2]. 

The usage of nanocomposite-based coatings has opened several possibilities in functional and high-performance textiles. Different metal-oxide (e.g., copper oxide, zinc oxide, and titanium dioxide) and metal (e.g., gold, zinc, copper, and silver) nanoparticles (NPs) have received significant attention as promising antimicrobial agents. These NPs possess superior action due to the higher surface-area-to-volume ratio, inducing their antimicrobial action via multiple mechanisms, namely by the direct interaction with the bacterial cell wall, inhibition of the biofilm formation, activation of the intrinsic and adaptive host immune responses, generation of reactive oxygen species (ROS) and interaction with intracellular components (e.g., DNA and proteins) [3,4,5,6]. In this respect, silver nanoparticles (AgNPs) have presented interesting antimicrobial properties, even in low concentrations [7,8].

Several techniques have been used to formulate textiles with AgNPs: the in situ thermal reduction, sonication, padding, dip-coating, spray, exhaustion, layer-by-layer, and electrospinning. However, numerous studies reported the potential uncontrolled leaching behavior of AgNPs, becoming a relevant environmental and health problem [9,10,11]. There is a need for new strategies to increase the stability of AgNPs on the fabrics.

Polyester (PES) fabrics have been largely used in various industries owing to their excellent strength, chemical resistance, processability, quick-drying and dimensional stability. Nevertheless, it presents a hydrophobic surface, where microorganisms can proliferate due to the abundant adsorption of metabolic products from the skin sweat/sebaceous glands [12]. The functionalization of PES with AgNPs can avoid the problems related to the microorganism’s proliferation, but the strategies for AgNPs deposition rarely promote acceptable adhesion of the AgNPs due to the absence of functional groups on the PES structure [13,14]. Surface modification techniques have been applied to introduce other chemical groups onto the PES surface, namely photo-induced irradiation, electron beam irradiation, enzymatic modification, alkaline hydrolysis, aminolysis, alcoholysis, and plasma treatments [12,15,16]. However, most of the accessible methods for stabilizing the AgNPs on PES require various functionalization steps, final treatments, drying, and/or curing processes. Each step increases time and cost, hindering large-scale production. Embedding NPs on the fiber polymeric matrix or reducing metallic salts to NPs in the bulk polymeric matrix are presented as high-performance methods. However, enveloping the particles in the fiber core significantly compromises their antimicrobial performance. Another developed strategy is the application of a binder to improve the adhesion of NPs onto textile substrates. Though, few reports were found in the literature using PES [17,18]. Enhancing the adhesion strength between the NPs and the PES fibers’ surface is imperative to ensure an efficient antimicrobial action, durability and avoid the undesirable release of metal NPs and ions [19].

Chitosan is an interesting biopolymer due to its inherent antimicrobial properties, biodegradability, non-toxicity, blood coagulating efficiency, antistatic features, and biocompatibility. It has been commonly studied for textile functionalization, namely as a binder for pigment printing, cationization of cotton, antimicrobial, anti-odor, and crease-resistant finishing [20,21,22]. In particular, metals easily interact with chitosan through electrostatic and chemical forces due to the presence of hydroxyl and amine groups [23]. Additionally, the combined antimicrobial effect of chitosan and metals have been explored to prepare novel nanocomposite materials with improved antimicrobial properties [24]. 

Organosilicon compounds have received more attention as coating agents due to their lack of toxicity, environmental friendliness, abrasion resistance, and physiological inertia [25]. Additionally, organosilicon compounds, including hexamethyldisiloxane (HMDSO), have been shown to tune the adhesion properties of a surface by exhibiting methyl groups within the silicon-organic matrix [26,27].

Herein, a fast and cost-effective method was developed to functionalize PES fabric with AgNPs via spray coating. Chitosan or HMDSO were sprayed in different layers, before and after the AgNPs deposition, to promote the adhesion of NPs onto the textile, to protect AgNPs from further oxidation, and to control the AgNPs release. In the AgNPs dispersion preparation, commercial polyvinylpyrrolidone-AgNPs (20.0–30.0 nm in size) were used. They were redispersed in ethanol and characterized by dynamic light scattering (DLS) and zeta potential. The textile samples were characterized by scanning electron microscopy (SEM) and X-ray photoelectron spectroscopy (XPS). The antimicrobial activity of textiles was evaluated against *Staphylococcus aureus* and *Escherichia coli*. Finally, the cytotoxicity of PES composites was assessed in HaCaT cells by the neutral red uptake assay.

## 2. Materials and Methods

### 2.1. Materials

Commercial pre-washed PES fabric (weight per unit area of 100 g⋅m^−2^) was used. The fabric was washed using a non-ionic detergent (1.0 g⋅L^−1^) at 60 °C for 60 min., rinsed with distilled water, and dried at 40 °C. Commercial spherical polyvinylpyrrolidone-coated (PVP) AgNPs 99.95%, with sizes of 20–30 nm, were purchased from SkySpring Nanomaterials Inc, Houston, TX, USA. Chitosan, Chito Clear 42,030– 800 CPS, was purchased from Primex, Siglufjordur, Iceland. Ethanol, acetic acid, nitric acid, HMDSO, Neutral red (NR) solution, and Triton™ X-100 detergent solution were purchased from Sigma-Aldrich, Taufkirchen, Germany. Dulbecco’s modified Eagle’s medium (DMEM) with 4.5 g⋅L^−1^ glucose and GlutaMAX™, fetal bovine serum (FBS), antibiotic (10,000 U⋅mL^−1^ penicillin, 10,000 µg⋅mL^−1^ streptomycin), Hanks’ balanced salt solution (HBSS) without calcium and magnesium [HBSS (-/-)] and 0.25% trypsin⋅1 mM^−1^ EDTA were obtained from Gibco^TM^, Thermo Fisher Scientific, Waltham, MA, USA. All the reagents used were of analytical or of the highest purity grade available.

### 2.2. Preparation of AgNPs Dispersions

All materials were previously cleaned with nitric acid (10% (*v*/*v*)) and rinsed with distilled water. Then, PVP-AgNPs were dispersed in ethanol (1.0 mg⋅mL^−1^) using an ultrasonic bath (30 min, 40 Hz) and ultrasound tip (15 min, 20 Hz). 

### 2.3. Formulation of PES Composites by Spray

**Figure 1:**
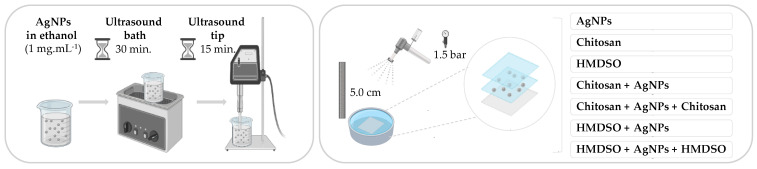
Schematic representation of the methodology adopted (images adapted from https://smart.servier.com (3 February 2022). Servier Medical Art by Servier is licensed under a Creative Commons Attribution 3.0 Unported License).

The AgNPs, chitosan solution (0.25% (*w*/*v*) of chitosan in 1% (*v*/*v*) acetic acid) and HMDSO layers were applied in both sides of the PES samples (10 × 10 cm^2^) via spray system, pressurized at 1.5 bar with a distance of 5 cm. Samples with different formulations were prepared: (i) only with AgNPs; (ii) HMDSO + AgNPs; (iii) HMDSO + AgNPs + HMDSO; (iv) chitosan + AgNPs; (v) chitosan + AgNPs + chitosan; vi) only HMDSO; (vii) only chitosan (Figure 1).

### 2.4. Washing Fastness

The washing fastness was assessed by performing 5 washing cycles (WC) according to EN ISO 15797 in a Datacolor Ahiba Lab Dyeing Machine (Lawrenceville, NJ, USA) at 75 °C, 40 rpm, for 15 min using a non-ionic surfactant (1.0 g⋅L^−1^) in a liquor bath ratio of 1/30 (*v*/*v*) [28]. 

### 2.5. Dynamic Light Scattering (DLS) Analysis

The size and zeta potential of PVP-AgNPs in the dispersion were measured using a Zeta Sizer-Nano (Malvern Instruments, Malvern, UK). Data were collected after 30 scans at 25 ± 1 °C, and zeta potential was measured in a moderate electrolytic concentrated solution.

### 2.6. Scanning Electron Microscopy (SEM)

Morphological analyses were carried out with an ultra-high-resolution FEG-SEM, NOVA 200 Nano, FEI Company (Hillsboro, OR, USA). Secondary electron images were performed with an acceleration voltage of 5 kV. Backscattering electron images were realized with an acceleration voltage of 15 kV. Samples were coated with an Au-Pd (20–80 weight %) film using a high-resolution sputter coater, 208 HR Cressington Company (Watford, UK), coupled to an MTM-20 Cressington High-Resolution Thickness Controller.

### 2.7. X-ray Photoelectron Spectroscopy (XPS)

Detailed surface atomic composition and bonding environment research was conducted employing XPS PHI-TFA spectrometer (Physical Electronics Inc., Chanhassen, MN, USA) equipped with an Al- monochromatic (7 mm) X-ray source operating at pass energy equal 1486.6 eV, with active surface charge neutralization. Data acquisition was performed with a vacuum better than 1 × 10^−8^ Pa. Spectra have been corrected to give the adventitious C 1s spectral component (C–C, C–H) binding energy of 284.5 eV. Spectra were analyzed for elemental composition using Multipack software.

Deconvolution into sub-peaks was performed by OriginLab software, using the Gaussian fitting function and Shirley-type background subtraction. No tailing function was considered in the peak fitting procedure.

### 2.8. Evaluation of Antibacterial Properties of PES Samples

Antibacterial testing was performed according to the ASTM-E2149 standard for the determination of the antimicrobial activity of antimicrobial agents under dynamic contact conditions. The tests were performed immediately after sample preparation with slight modifications. Both Gram-positive and Gram-negative bacteria were used, respectively *Staphylococcus aureus* (American Type Culture Collection (ATCC 25923) and *Escherichia coli* (*E. coli*, ATCC 25922). The pre-inoculum of each bacterium was prepared in tryptic soy broth (Merck) and after 12 h of incubation at 37 °C and 120 rpm, the inoculum of each bacterium was centrifuged, the supernatant was eliminated, and the bacteria washed with sterile phosphate buffer saline (PBS). Then, the concentration of each bacterium was adjusted to 2 × 10^7^ CFU⋅mL^−1^. PES samples (1 × 1.5 cm) were inoculated in 5 mL of bacterial suspension for 3 h and 5 h at 37 °C and 120 rpm. Afterward, aliquots of these suspensions were collected and used to prepare 10-fold serial dilutions, which were cultured on agar plates for the determination of viable cells. The number of colony-forming units (CFUs)/mL was established before (0 h) and after (3 h and 5 h) contact with the fabrics. The results were expressed as log reductions, calculated as the ratio between the number of surviving bacteria colonies present on the tryptic soy agar (TSA) plates, before and after contact with the fabric. Antibacterial studies were performed in triplicate in two independent experiments.

### 2.9. Cytotoxicity

The cytotoxicity of the PES composites was evaluated in HaCaT cells, an immortalized human keratinocyte cell line, after 24 h after exposure, by the NR uptake assay. 

HaCaT cells were routinely cultured in 75 cm^2^ flasks using DMEM with 4.5 g·L^−1^ glucose and GlutaMAX™, supplemented with 10% of FBS, 100 U·mL^−1^ of penicillin, and 100 μg·mL^−1^ of streptomycin. Cells were grown at 37 °C, in a 5% CO_2_-95% air atmosphere, and the medium was changed every 2 days. At 80–90% of confluence, cells were detached from the culture flasks via trypsinization (0.25% trypsin·mM^−1^ EDTA). The cells were seeded in 96-well plates at a density of 20,000 cells/well. Freshly prepared extracts of each sample (previously hermetically sealed and sterilized in an autoclave at 121 °C, 1.2 bar for 20 min.) were used in the evaluation of cytotoxicity, accordingly with ISO 1993-5 (Biological evaluation of medical devices—Part 5: Tests for in vitro cytotoxicity) [29]. Briefly, the extraction was performed in a complete cell culture medium, at a proportion of 0.1 g·mL^−1^ (ratio recommended for textiles), in a sterile, chemically inert, and closed container, for 24 ± 2 h, at 37 ± 1 °C, and under agitation. The extract was then directly used (100% concentration) or diluted in fresh cell culture medium at different concentrations (2, 4, 8, 16, and 32-times dilutions leading to 50, 25, 12,5, 6.25, and 3.125% concentrations). After 24 h seeding, the cells were exposed to the extracts of the medical devices (0–100%) for another 24 h. Extraction cell culture medium (without the test material) was also submitted to the same extraction conditions and used as a control. Triton™ X-100 (1% (*v*/*v*)) was used as positive control. The cells used in all experiments were taken between the 45th and 50th passages. 

### 2.10. Neutral Red (NR) Uptake Assay

The cytotoxicity of the PES samples was assessed by the NR uptake assay that, based on the capacity of living cells to incorporate and retain the supravital dye NR within the lysosomes, provides a quantitative estimation of the number of viable cells in the culture. After 24 h exposure to the extracts of the samples, the cell culture medium was aspirated and a fresh cell culture medium containing NR (50 μg·mL^−1^) was added. Cells were then incubated for 90 min, at 37 °C, in a humidified 5% CO_2_-95% air atmosphere. After incubation, the cell culture medium was removed, and the NR dye retained only by viable cells was extracted (absolute ethyl alcohol/distilled water (1:1) with 5% (*v*/*v*) acetic acid). The absorbance was subsequently measured, at 540 nm, in a multi-well plate reader (PowerWaveX BioTek Instruments, VT, Santa Clara, CA, USA). The percentage of NR uptake relative to that of the control cells (0%) was used as the cytotoxicity measure. Four independent experiments were performed in triplicate. 

### 2.11. Statistical Analysis

All statistical calculations were performed using the GraphPad Prism 8 for Windows (GraphPad Software, San Diego, CA, USA). The normality of the data distribution was assessed using the KS, D’Agostino & Pearson omnibus, and Shapiro–Wilk normality tests. One-way analysis of variance (ANOVA) was used to perform the statistical comparisons, followed by Dunnett’s multiple comparisons test. Details of the performed statistical analysis are described in the figure captions. Differences were considered significant for *p* values lower than 0.05.

## 3. Results and Discussion

### 3.1. PES Fabrics Functionalization and Characterization

The present research focuses on the stabilization of AgNPs on PES fabrics using a biopolymer, the chitosan, and an organosilicon compound, HMDSO, to improve the stability of the AgNPs and control antibacterial efficacy. PES nanocomposites were prepared by spray deposition of AgNPs and chitosan or HMDSO layers. The following structures were prepared: (i) chitosan + AgNPs, (ii) chitosan + AgNPs + chitosan, (iii) HMDSO + AgNPs, and (iv) HMDSO + AgNPs + HMDSO. Control samples were also prepared by loading the fabric only with AgNPs, HMDSO, or chitosan (Figure 1). The AgNPs dispersion was characterized by DLS and zeta potential. The textile samples were characterized by SEM and XPS. Furthermore, the nanocomposites were washed 5 times to predict the release of AgNPs and the effect of the chitosan or HMDSO layers. The differences between washed and unwashed samples were detected by XPS analysis and confirmed via antimicrobial testing. The HMDSO and chitosan layers were meant to delay the AgNPs oxidation to a more controlled and durable release of the Ag ions. Especially after several washing cycles. 

In the first step, commercially available AgNPs were redispersed using an ultrasound bath and ultrasound tip in absolute ethanol. The AgNPs dispersion was assessed by DLS measurements showing an average size of 281.0 ± 1.5 nm, a polydispersity index of 0.10 ± 0.02, and suitable colloidal stability with a zeta potential value of −31.0 ± 1.2 mV. 

**Figure 2:**
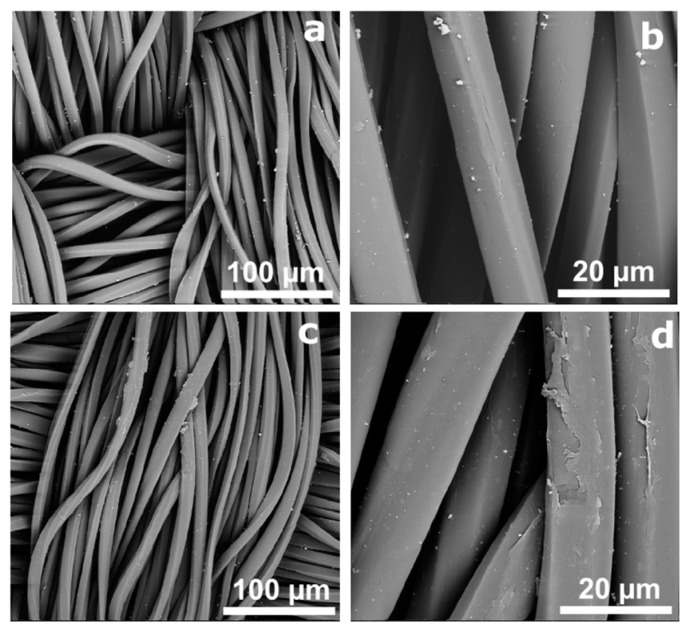
SEM micrographs of PES fabric with AgNPs (**a**,**b**) and with a final chitosan layer (chitosan + AgNPs + chitosan, (**c**,**d**) at magnifications of ×1000 and ×5000.

In the second step, the PES formulations were obtained by a spray method, where the AgNPs distribution onto the fabric and corresponding morphology were evaluated by SEM (Figure 2). The SEM images confirm the presence of AgNPs in all samples with no significant differences in their distribution or morphology, even in samples with an initial layer of chitosan or HMDSO, which may be explained by the successful utilization of the spray method for NPs deposition. The distance and the velocity of the spray flow were the same in all samples. The AgNPs onto PES showed uniform distribution, some agglomeration in all samples, and a quasi-spherical structure. In samples with a final layer of chitosan, it was possible to observe that the available AgNPs linked to the fabrics’ surface were mostly covered by chitosan (some internal areas were uncovered). It was not possible to detect the distribution of HMDSO by SEM. No visual differences were found in samples containing HMDSO in the performed magnifications.

**Figure 3:**
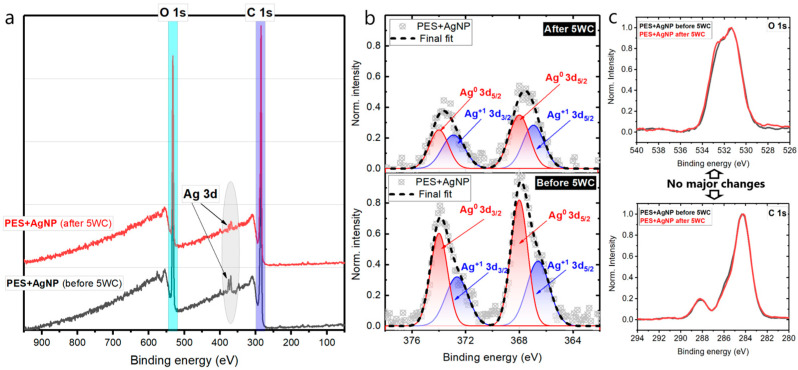
(**a**) Survey spectra of control PES loaded with AgNPs before and after five consequent washing procedures. (**b**) Gaussian deconvolution of high-resolution XPS Ag 3d core levels peaks. (**c**) O 1s and C 1s high-resolution spectra.

Chemical composition analysis of the unprotected AgNPs distributed over a control PES substrate surface was investigated before and after washing (Figure 3a). An evident spin-orbit doublet between 366–376 eV is observed, and it is attributed to Ag 3d core levels confirming the presence of noble nanoparticles on both PES substrates, before and after washing, respectively. However, after repetitive washing cycles, the intensity of silver-related peaks undergoes a relative decrease (Figure 3a). Deconvolution also confirms that the area under a final fit (black dashed curves) before washing is about 1.79 times larger than after washing (Figure 3b). It can mean the only fact that an unprotected nanosilver has been partially rinsed off during laundry. The oxidation states configuration of the remaining silver differs from that observed for the non-washed sample. By using the formula:

(1) $$Ag\left(0\right)=\frac{Ag^{0}}{Ag^{0}+Ag^{1+}}$$

and considering peak areas, it can be estimated that a portion of metallic silver of non-treated sample is larger (59%) than the washed one (52%). Note that neither carbon C 1s nor oxygen O 1s peaks related to PES substrate have not been considerably modified, proving its stability under multiple laundry cycles (Figure 3c). The oxygen/carbon atomic ratio remains unchanged as well, being equal to 0.30 in both cases.

**Figure 4:**
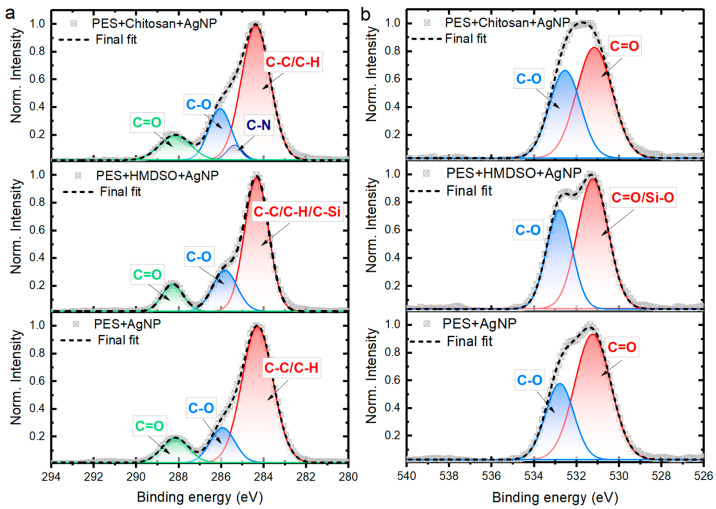
(**a**) H igh-resolution C 1s peak accompanied by Gaussian components deconvolution. (**b**) The same elaboration for O 1s spectra of the indicated samples.

Aiming to protect the chemical environment of AgNPs, and especially to persist their population on PES substrate, two different sandwich-like spray-coated protective polymer layers were investigated (HMDSO and Chitosan). C 1s and O 1s spectra were elaborated to monitor the presence of HMDSO and chitosan protections after spraying. Considering PES oxygen/carbon ratio stability before and after washing, this feature can serve as a “presence indicator” of HMDSO and chitosan on top of PES. This parameter is different for each case and was found to be 0.30, 0.32, and 0.34 for PES, HMDSO, and chitosan, respectively. Slightly higher oxygen content is observed for chitosan coating, and the lowest is attributed to silver decorated PES sample. This finding denotes a good correlation with the Gaussian deconvolution, where a contribution of the C-O surface component, defined by a peak area, increases following the oxygen/carbon atomic ratio. A calculated area enclosed under the “blue” peak given in arbitrary units is as follows: PES = 0.37, HMDSO = 0.43, chitosan = 0.49 (Figure 4a).

Similarly, the C-O component behaves within recorded O 1s spectra. The integrated area of the peak located around 532.7 eV (blue peak) increases its contribution into a final fitted curve (Figure 4b). The numbers expressed in shares (%) are 32%, 38%, and 41% for PES, HMDSO, and chitosan, respectively. These observations confirm a successful coating of PES by HDMSO and chitosan protective layers.

**Figure 5:**
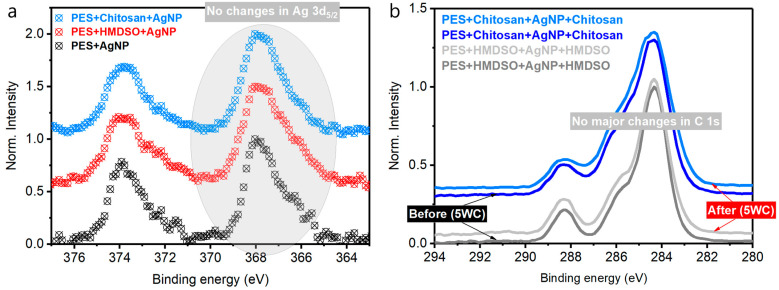
(**a**) XPS cut-off representing Ag 3d electronic orbital binding energy range. (**b**) The effect of washing cycles on high-resolution C 1s spectra of protective polymer coatings.

Further, a new portion of nanosilver was attached to freshly created PES + HDMSO and PES + chitosan samples. Collected XPS results related to Ag 3d_5/2_ core levels revealed a high level of similarity in shape for all three unwashed samples, indicating no major alterations in oxidation shares between Ag(0) and Ag(+1) (Figure 5a). The presence of Ag(+1) can be related to the oxidation of the metallic silver in contact with air to AgOH, which consequently decomposes to Ag_2_O [30]. After completing a sandwich-like structure by depositing a second protective layer made of the same polymer, noble metal NPs were not detected by XPS, additionally proving the efficiency of a spray-coating technique. The influence of washing cycles was tested again for PES + HDMSO + AgNP + HMDSO and PES + chitosan + AgNP + chitosan. Following the XPS data, neither the shape nor peak intensity of C 1s was altered. Additionally, the oxygen/carbon atomic ratio remained unchanged (Figure 5b). Additionally, a typical small Si 2p peak for HMDSO and N 1s peak for chitosan remained with similar features before and after laundry, indicating superior stability of sprayed protective layers.

### 3.2. Evaluation of Antibacterial Properties of PES Samples

**Figure 6:**
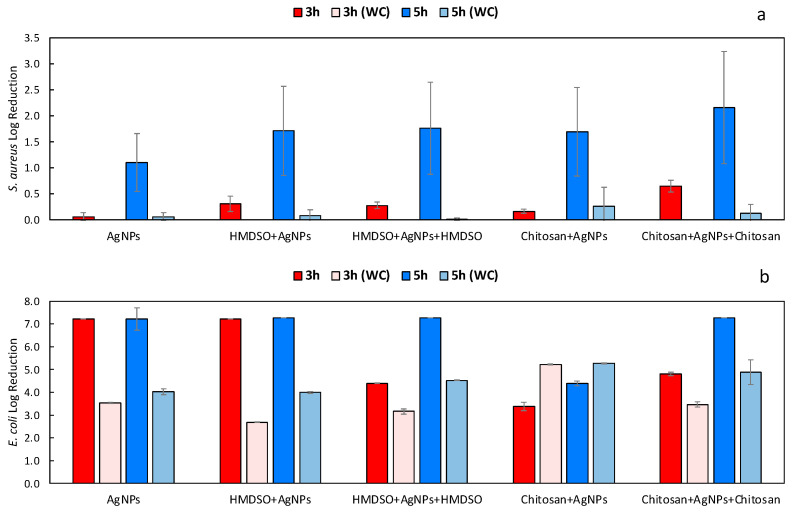
Antimicrobial action of samples against *S. aureus* (**a**) and *E. coli* (**b**) after 3 h and 5 h of contact, before and after 5 WC.

The antibacterial activity of PES samples functionalized with AgNPs and chitosan or HMDSO, as well as the corresponding control samples, were tested against *S. aureus* and *E. coli* by shake flask method (Figure 6). The tests were performed at different time points, after 3 h and 5 h of contact, to evaluate the time-kill kinetics of the AgNPs activity and the effect of the different layers of each configuration. The antimicrobial effect of the samples, before and after 5 WC, was also tested to assess the AgNP’s endurance.

The composites exhibited low antimicrobial efficacy against *S. aureus* and strong activity against *E. coli*. This can be justified by the differences in the structure of the cell walls of the two types of bacteria, once *S. aureus* presents a thicker peptidoglycan layer (30 nm thickness) than the thinner structure of the *E. coli* cell wall (~3–4 nm thickness). Thus, the probability of the positively charged AgNPs being immobilized in the negative and thicker peptidoglycan layer of *S. aureus* bacteria is much higher than in *E. coli*. This suggests that the antimicrobial effect is controlled by the capability of the silver ions and AgNPs to disrupt the bacterial cell wall [31,32,33].

Generally, the results after 5 h of contact are equal or higher than the results after 3 h of contact but the layers showed to promote different behaviors in the AgNPs action. Starting from the results against *S. aureus*, after 5 h of incubation, the sample with 2 layers of chitosan (chitosan + AgNPs + chitosan) showed the highest log reduction (2.15 ± 1.08). Despite the higher exposition of the AgNPs in other samples, data suggests a potential synergistic effect to have taken place between chitosan and AgNPs. Some studies have been performed using chitosan to improve the adhesion of metal NPs onto textiles, while improving their inherent features, such as antimicrobial activity (chitosan and NPs) [34]. However, these studies mostly focus on cotton fabrics and no examples were found using PES. Chitosan has been described as a bacteriostatic polymer. Chitosan can interact with the cell membrane of pathogens once it presents a negative surface charge, reducing cell permeability to important environmental factors necessary to their viability. It can also interact with DNA, form chelates with microorganisms’ nutrients, and form an intense film on the cell’s surface, inhibiting their growth [35]. The remainder tested samples presented similar results after 5 h of contact with log reduction between 1.10 and 1.76. The results after 3 h of contact and after 5 WC did not show any relevant antibacterial efficacy against *S. aureus*. 

The results against *E. coli* provided more information about the layer’s efficacy. The control sample with just AgNPs showed an analogous log reduction after 3 h and 5 h of contact (7.22). A similar log reduction was obtained in the sample with an initial layer of HMDSO (HMDSO + Ag), 7.22 and 7.27 after 3 h and 5 h of contact. After 5 WC, the control sample exhibited 3.53 of log reduction and the HMDSO + AgNPs sample presented 2.69 after 3 h of incubation, and 4.09 and 3.99 after 5 h, respectively. These results suggest that only one layer of HMDSO does not provide any improvement in AgNPs adhesion over the control. However, when a final layer of HMDSO was added (HMDSO + AgNPs + HMDSO), the sample presented a slower antimicrobial effect, with a log reduction of 4.40 after 3 h of incubation (instead of 7.22) and 7.27 after 5 h. Again, after washing a slower antimicrobial effect was observed. This sample showed a log reduction of 3.16 after 3 h and a log reduction of 4.53 after 5 h, showing a superior result than the control and the HMDSO + AgNPs samples.

Since the amount of AgNPs in the unwashed samples is the same (spray application), the reduction in activity in the first hours of contact when a final layer of HMDSO is present is due to the greater stabilization of the AgNPs onto the fabric but also to the superior protection against the oxidation. The mechanism of action of AgNPs against *E. coli* have shown to be closely related to the release of silver ions by (i) oxidative stress caused by ROS, (ii) interaction of silver ions with thiol groups in proteins, and (iii) the destruction of the bacteria cells via strong affinity between silver ions and cell membrane. The release of silver ions and the ROS generation have been widely induced when Ag_2_O is present [36,37]. In this work, the used AgNPs have an average diameter of 20–30 nm, and after immobilization form bigger agglomerated clusters that cannot enter inside the bacteria. Thus, the antibacterial effect seems mainly to be promoted by the ions release.

When using chitosan different effects were observed. The adhesion of AgNPs onto the fabric was superior using an initial layer of chitosan. The sample chitosan + AgNPs displayed a log reduction of 3.37 and 4.40 after 3 h and 5 h of contact, respectively. These results demonstrated the protective effect of chitosan over AgNPs and their superior adhesion to the substrate. However, after 5 WC, the same sample presented a superior antimicrobial effect, 5.22 and 5.27 of log reduction after 3 h and 5 h of contact, respectively. This can be attributed to a dual effect: a better adhesion of AgNPs and to an increased oxidation after washings as proved in the XPS results. Moreover, when the AgNPs layer was deposited over the first layer of chitosan, the chitosan was dissolved and the AgNPs were wrapped in the chitosan, justifying the inferior antimicrobial effect before washings. After washings, some chitosan was removed, decreasing the protective effect under AgNPs, the exposition of AgNPs increased, and consequently, the antimicrobial action also increased. Following this evidence, the inferior but suitable antimicrobial data of the chitosan + AgNPs sample before washing and the superior antimicrobial effect after washing should be attributed to the superior adhesion and protection of AgNPs using the initial layer of chitosan. Lastly, when a final layer of chitosan was added, a synergistic antimicrobial effect was also observed between chitosan and AgNPs. Despite the higher AgNPs exposition in the chitosan + AgNPs sample, the samples chitosan + AgNPs + chitosan showed a higher antimicrobial effect after 3 h of incubation (4.81 of log reduction) and 5 h of incubation (7.27 of log reduction). After WC, the antimicrobial results were also relevant, but the synergism was not that evident reporting log reductions of 3.47 and 4.88 after 3 h and 5 h of contact, respectively. 

Although the results with chitosan layers after washing were comparable to the control samples just with AgNPs, by using chitosan it was possible to guarantee the washing fastness of the AgNPs (the AgNPs remained on the fabric). This strategy may prevent environmental contamination with heavy metals during the use and washing of antimicrobial textiles, enhance the durability of the antimicrobial effects, and protects the users from unnecessary exposure to AgNPs and, consequently, their cytotoxicity.

### 3.3. Evaluation of the Cytotoxicity of PES Samples Extracts

**Figure 7:**
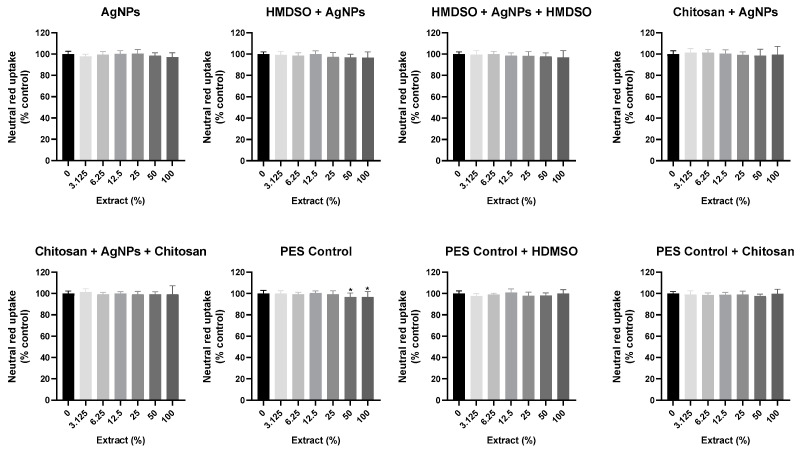
Cytotoxicity of the PES samples extracts (0–100%) evaluated in HaCat cells by the NR uptake assay, after 24 h exposure. Results are expressed as mean ± standard deviation (SD) from 4 independent experiences, performed in triplicate. Statistical comparisons were made using one-way ANOVA followed by Dunnett’s multiple comparisons tests (* *p* < 0.05 vs. 0%).

The cytotoxicity of the extracts of the PES samples was assessed after 24 h exposure, by the NR uptake assay. For that purpose, HaCaT keratinocyte-like cells were used as an in vitro model. No significant effects on NR uptake were detected after 24 h exposure to the extracts of AgNPs, HMDSO + AgNPs, HMDSO + AgNPs + HMDSO, chitosan + AgNPs, chitosan + AgNPs + chitosan, PES control + HDMSO and PES control + chitosan, at all the tested concentrations (0–100%) (Figure 7). For PES control extract, a small but significant reduction in NR uptake was observed for the highest tested concentrations (NR uptake significantly decreased to 96.74 and 96.75, 24 h after exposure to 50 and 100% of PES control extract, respectively, and when compared to control cells (0%)). Noteworthy, and accordingly with the ISO 1993-5, the medical devices under study are considered non-cytotoxic as the relative cell viability observed for the highest concentration of the sample extract (100% extract) was always higher than 70% when compared to the control cells (0%) [29].

## 4. Conclusions

This research envisaged the development of PES nanocomposites with controlled antimicrobial performance against *S. aureus* and *E. coli*, using AgNPs and chitosan or HMDSO layers. The chitosan or HMDSO layers were applied on PES fabric before and/or after AgNPs deposition. The samples were successfully prepared by spray coating and 5 WC were conducted after deposition. Successful PES functionalization and AgNPs content were verified by XPS and SEM analyses. The antimicrobial results showed that just an initial layer of HMDSO does not improve the AgNPs adhesion. However, when an initial and final layer of HMDSO was applied, AgNPs were stabilized onto PES fabric and the treatment prevented the complete loss of AgNPs during the washings. When chitosan was used, different results were obtained. With only one layer of chitosan, the adhesion of AgNPs to the PES fabric was significantly improved. Moreover, when an initial and final layer of chitosan was added, a controlled antimicrobial action was attained, and synergistic antimicrobial effects were evidenced between chitosan and AgNPs. Here, too, superior washing fastness was observed. Lastly, cytotoxicity studies showed the biocompatibility of the prepared PES nanocomposites. 

These nanocomposites will open new perspectives for the use of AgNPs to PES functionalization in the healthcare sector with minimal environmental contamination during the use and washing of antimicrobial textiles, superior durability, and controlled antimicrobial effect.

## Data Availability

Not applicable.
